# Measuring the effects of a personalized music intervention on agitated behaviors among nursing home residents with dementia: design features for cluster-randomized adaptive trial

**DOI:** 10.1186/s13063-021-05620-y

**Published:** 2021-10-07

**Authors:** Ellen M. McCreedy, Roee Gutman, Rosa Baier, James L. Rudolph, Kali S. Thomas, Faye Dvorchak, Rebecca Uth, Jessica Ogarek, Vincent Mor

**Affiliations:** 1 Center for Gerontology & Healthcare Research, Brown University School of Public Health, 121 South Main St., Box G-S121-6, Providence, RI 02912 USA; 2 Department of Health Services, Policy & Practice, Brown University School of Public Health, 121 South Main St., Providence, RI 02912 USA; 3 Center for Long-Term Care Quality & Innovation, Brown University School of Public Health, 121 South Main St., Providence, RI 02912 USA; 4 Department of Biostatistics, Brown University School of Public Health, 121 South Main St., Providence, RI 02912 USA; 5 U.S. Department of Veterans Affairs Medical Center, 830 Chalkstone Ave., Providence, RI 02908 USA

**Keywords:** Embedded pragmatic trials, Adaptive trials, NIH Stage Model for Behavioral Intervention Development, Statistical imputation

## Abstract

**Background:**

Agitated and aggressive behaviors (behaviors) are common in nursing home (NH) residents with dementia. Medications commonly used to manage behaviors have dangerous side effects. NHs are adopting non-pharmacological interventions to manage behaviors, despite a lack of effectiveness evidence and an understanding of optimal implementation strategies. We are conducting an adaptive trial to evaluate the effects of personalized music on behaviors. Adaptive trials may increase efficiency and reduce costs associated with traditional RCTs by learning and making modifications to the trial while it is ongoing.

**Methods:**

We are conducting two consecutive parallel cluster-randomized trials with 54 NHs in each trial (27 treatment, 27 control). Participating NHs were recruited from 4 corporations which differ in size, ownership structure, geography, and residents’ racial composition. After randomization, there were no significant differences between the NHs randomized to each trial with respect to baseline behaviors, number of eligible residents, degree of cognitive impairment, or antipsychotic use. Agitated behavior frequency is assessed via staff interviews (primary outcome), required nursing staff conducted resident assessments (secondary outcome), and direct observations of residents (secondary outcome). Between the two parallel trials, the adaptive design will be used to test alternative implementation strategies, increasingly enroll residents who are likely to benefit from the intervention, and seamlessly conduct a stage III/IV trial.

**Discussion:**

This adaptive trial allows investigators to estimate the impact of a popular non-pharmaceutical intervention (personalized music) on residents’ behaviors, under pragmatic, real-world conditions testing two implementation strategies. This design has the potential to reduce the research timeline by improving the likelihood of powered results, increasingly enrolling residents most likely to benefit from intervention, sequentially assessing the effectiveness of implementation strategies in the same trial, and creating a statistical model to reduce the future need for onsite data collection. The design may also increase research equity by enrolling and tailoring the intervention to populations otherwise excluded from research. Our design will inform pragmatic testing of other interventions with limited efficacy evidence but widespread stakeholder adoption because of the real-world need for non-pharmaceutical approaches.

**{2a} Trial registration:**

ClinicalTrials.govNCT03821844. Registered on January 30, 2019. This trial registration meets the World Health Organization (WHO) minimum standard.

**Supplementary Information:**

The online version contains supplementary material available at 10.1186/s13063-021-05620-y.

## {6a} Introduction

Most people with dementia will manifest agitated and/or aggressive behaviors (behaviors) at some point during their disease [1]. These behaviors are a significant source of patient and caregiver distress and can precipitate placement in a nursing home (NH) [2]. In addition to decreasing the quality of remaining life for NH residents with dementia, behaviors can result in injury to other residents [3] and increased staff burnout [4]. Antipsychotic medications, often used to manage such behaviors, increase the risk of death in people with dementia [5]. To improve dementia care, there is a need to identify effective non-pharmaceutical interventions that improve behaviors.

One popular non-pharmaceutical intervention is Music & Memory^SM^ (M&M). M&M is a personalized music program in which the music a resident liked as a young adult is loaded onto a personal music device and administered by NH staff to address agitation [6]. While the mechanism of action is unknown, evidence suggests early musical memories are stored in a part of the brain affected later in dementia [7]. Listening to music may elicit autobiographical memories [8–10] and evoke a relaxation response [11, 12]. We hypothesize behaviors resulting from social isolation, depression, confusion, or sensory deprivation [13] may be affected by M&M.

The need for non-pharmaceutical approaches for managing behaviors in residents with dementia has resulted in widespread adoption of M&M ahead of effectiveness evidence. The largest pragmatic, randomized trial of the program to date enrolled 59 residents with dementia from 10 NHs and found no significant decrease in agitation after exposure compared to usual care controls [14]. Weaknesses of that study include small sample size, lack of a measure of behaviors close in time to the intervention, and inadequate implementation (music was only used an average of 9 days a month) [14].

Our study addresses the limitations of previous studies by enrolling over 1200 NH residents from 81 NHs, directly observing residents close in time to delivery of the intervention, and by using an adaptive trial design to test alternative implementation strategies which may improve nursing staff uptake of the intervention. Adaptive trials can increase efficiency and reduce costs associated with traditional RCTs by learning and making modifications to the trial while it is ongoing [15].

{7, 8, 6b} This protocol describes two parallel cluster-randomized, superiority trials designed to test the effectiveness of a personalized music intervention on agitated behaviors among nursing home residents with dementia compared to usual nursing care for behaviors (an appropriate comparator for a pragmatic trial). {7} We will also describe how the adaptive design will be used to test alternative implementation strategies, increasingly enroll residents who are likely to benefit from the intervention, and seamlessly conduct a stage III/IV trial [16]. To our knowledge, this is the first cluster-randomized controlled trial to use an adaptive design.

## Methods

Methods are reported using SPIRIT guidelines (see Additional file 1 for the checklist) [17].

### Participants

Potentially eligible NHs from four partnering NH corporations were identified and allowed to opt in.

{10} NHs were potentially eligible if they had at least 20 residents who were long-stay (90 of the last 100 days spent in the NH), had a dementia diagnosis, and were not completely deaf. The number of eligible residents in a NH was determined using the Minimum Data Set (MDS) [11]. MDS data are derived from routine, standardized assessments of residents. These data are similar to electronic health record data that can be used to identify study-eligible patients in large embedded pragmatic trials or quality improvement programs [18].

NH leadership removed potentially eligible NHs with competing demands that would affect successful implementation, including a recent poor inspection or major leadership change. NHs with prior exposure to M&M were also removed. Priority was given to NHs located in a common geographic area to reduce data collection costs. There were 44 potentially eligible NHs for corporation A, 15 for corporation B, 19 for corporation C, and 55 for corporation D. Most NH administrators were interested in participating in the trial; five declined. We enrolled NHs when they returned their letters of commitment, until each corporation reached capacity (A 24 NHs, B 12 NHs, C 15 NHs, D 30 NHs). Capacity was determined by corporation relative size and the desire to have the same number of NHs in each of the arms of the two parallel trials, where control facilities in the first trial are assigned to the treatment group in the second trial.

### Study settings

{9, 15} We elicited volunteer NH corporations via the American Health Care Association and approached four NH corporations to participate to assure diversity in size, ownership structure, geography, and residents’ racial composition (Table 1). Two for-profit corporations (one with fewer than 25 eligible NHs (small), one with more than 50 eligible NHs (large)) and two non-profit corporations (one small, one large) were recruited. The Midwestern corporations had predominantly white resident populations, and the mid-Atlantic and Southern corporations had higher proportions of African American residents (40–50%). The corporations also differed in baseline CMS 5-Star quality ratings [19], antipsychotic use, and percent of eligible residents with behaviors. A list of participating corporations can be found at clinicaltrials.gov.

**Table 1:** Characteristics of participating corporations and their potentially eligible nursing homes

	Corporations			
	A	B	C	D
**Characteristics of participating corporations**				
Eligible nursing homes (#)	44	15	19	55
Geographic region	Mid-West	Mid-West	Mid-Atlantic	South
Ownership type	Non-profit	Non-profit	For-profit	For-profit
**Characteristics of eligible nursing homes**	*Mean* (*SD*)	*Mean* (*SD*)	*Mean* (*SD*)	*Mean* (*SD*)
African American residents (%)	0.5 (0.9)	0.0 (0.0)	42.0 (20.4)	40.0 (27.4)
CMS 5-Star Quality Rating^a^	3.6 (1.1)	4.0 (1.1)	3.0 (1.5)	3.4 (1.3)
Residents with antipsychotic medication use in past 7 days (%)	16.3 (6.7)	12.2 (6.6)	25.2 (13.6)	17.3 (8.5)
Residents with any agitated or aggressive behaviors in past 7 days (%)	11.2 (7.2)	9. 4 (6.9)	21.6 (15.3)	11.6 (11.7)

*CMS* Centers for Medicare & Medicaid Services

^a^Score ranges from 1 to 5 stars, with five stars indicating the highest quality nursing homes

### Interventions

{11a} Music & Memory^SM^ is a personalized music program in which the music a resident with dementia liked as a young adult is loaded onto a personal music device and administered by NH staff to preempt or reduce agitation [6]. Earphones are used to deliver the personalized music to the residents. From a list of potentially eligible residents, NH staff choose 15 residents to receive the program. NH staff are instructed to use the music at times of day when behaviors were likely or at early signs of agitation. The recommended dose is 30 min a day. {11d} The control condition is usual care, which may include the use of ambient music or group music.

### Outcomes

{12} The primary study outcome is agitated behaviors. Agitated behaviors are measured in three ways: researcher-collected staff interviews of NH staff about resident behaviors in the past 2 weeks (primary study outcome); researcher-collected direct observation of residents (secondary outcome); and NH-collected standardized assessment data about resident behaviors in the past week (secondary outcome).

Researcher-collected behavior data include staff interviews (primary outcome) and direct observations (secondary outcome). {18a} Data collectors receive a 3-day intensive training and are required to have weekly phone calls with study staff while in the field. To collect the study primary outcome, the research staff interview a nursing staff member who knows the resident well using the Cohen-Mansfield Agitation Inventory (CMAI) [20], which asks about the frequency of 29 agitated behaviors in the past 2 weeks. Response options for each CMAI item range from never (1) to several times per hour (7). The total CMAI score ranges from 29 to 203. The total CMAI serves as the primary outcome variable in this study. Using the Agitation Behavior Mapping Instrument (ABMI) [21], research staff also observe residents for short intervals (3 min per observation) and record the number of times that 14 specific agitated behaviors occur (range 0–140). Each resident is observed at least four standardized times over the course of each 3-day visit. Both the ABMI and CMAI have been widely used in the NH setting and have high interrater reliabilities (0.88 to 0.93) [20, 21].

NH-collected behavior data captured in the MDS (secondary outcome) includes frequency of physical behavioral symptoms directed toward others, verbal behavioral symptoms directed toward others, other behavioral symptoms not directed toward others, and behaviors related to resisting necessary care [22]. These items are derived from the CMAI domains and include many of the same behaviors. Frequency in the past week is reported as behavior not exhibited, behavior occurred 1–3 days, behavior occurred 4–6 days, or behavior occurred daily. These four behavioral frequency items in the MDS are summed to create the Minimum Data Set Agitated and Reactive Behavior Scale (MDS-ARBS), which has adequate internal consistency [23].

There are several other secondary outcomes. MDS data measure changes in the administration of antipsychotic, anxiolytic, and hypnotic medications. Another secondary outcome of interest is resident mood. The Lawton Observed Emotion Rating Scale (OERS) measures researcher-observed pleasure, anger, anxiety/fear, sadness, and general alertness in NH residents with advanced dementia [24]. Depressed mood is also assessed using a version of the Patient Health Questionnaire (PHQ-9) [25] embedded in the MDS [25].

### Data collection, transfer, and monitoring

{19} On-site data will be collected using tablets through data entry systems developed in Qualtrics. Data will be uploaded to the Qualtrics central servers using a secure channel. {27} When entering the study data in Qualtrics, the patient data will only be identified by pre-assigned study identification numbers; no personally identifying information (PII) or existing identifiers (e.g., medical record number, social security number) will be entered. Partnering corporations will also transfer their MDS data to the research institution servers via a secure SFTP protocol with password protection. The information systems manager will be in charge of all data transfers, and he will replace PII with study identification numbers to allow linkage of data for analytic purposes. {29} Data use agreements limit access to participant-level analytic files to the study team. {31c} The full study protocol and statistical code will be made public through the Brown data repository (https://repository.library.brown.edu). {31a} Lay language results will be disseminated with partnering corporations and posted on the Brown University, Center for Long-Term Care Quality & Innovation public-facing website.

### Standardizing and monitoring implementation

{11c}This study had a 6-month pilot phase focused on developing and testing a step-by-step implementation guide [26]. The guide provides step-by-step guidance on identifying residents’ preferred music, downloading it onto a personalized music device, and testing and using the music with the resident.

All participating NHs receive two types of training. First, NH staff participate in standard M&M training and certification, which includes two 1.5-h live webinars describing the program. The second in-person training was developed by researchers during the pilot and is administered jointly by corporate leadership and study consultants. This training follows the steps outlined in the implementation guide. Staff required to attend the in-person trainings include the NH administrator, director of nursing, activities director, a nurse manager, and a certified nursing assistant.

Another aspect of the program implementation includes monthly coaching calls with the NHs to monitor progress, troubleshoot problems, and share successes. Monthly coaching calls are led by corporate trainers and study implementation consultants. Participation in monthly calls is tracked; calls are audio recorded.

Adherence is monitored using data from the personalized music devices. For each song on the device, these data document the length in minutes and a count of the times the song was played, yielding an estimate of the amount of exposure to the intervention for each resident.

### Interim analyses and stopping

{11b, 21b} There are no formal stopping rules for the two trials. The study may be discontinued at any time by the Institutional Review Board or the National Institute on Aging, as part of their duties to ensure that research participants are protected. {24, 26a}This was deemed a minimal risk study by the Brown University Institutional Review Board, which issued a waiver of individual consent (#1705001793). Given the short implementation period for each trial (8 months), interim analyses were not practical.

### Randomization

{16a, 16b, 16c} NHs were randomized within corporate strata. Within each corporation, NHs were partitioned into triplets based on the Mahalanobis distance from the overall mean [27] on percentage of eligible residents with any agitated or aggressive behavior and number of eligible residents. Balancing was important because behaviors vary considerably at the NH level because of resident composition, staffing, and the degree of “ascertainment” and documentation of agitated behaviors [26, 28], and NHs with more eligible residents can be more selective in who receives the intervention. Within balanced triplets, one NH was randomly assigned to either being in the treatment group in the first parallel trial, being in the control condition in the first parallel trial and treatment group in the second parallel trial, or being in control condition in the second parallel trial. Random assignment was performed by the study statistician (RG). After randomization, there were no significant differences between the NHs randomized to the three groups with respect to baseline behaviors, number of eligible residents, degree of cognitive impairment, or antipsychotic use (Table 2).

**Table 2:** Characteristics of nursing homes at baseline (post-randomization)

	Sequence 1 (*n* = 27 nursing homes)	Sequence 2 (*n* = 27 nursing homes)	Sequence 3 (*n* = 27 nursing homes)
	*Mean* (*SD*)	*Mean* (*SD*)	*Mean* (*SD*)
**Resident composition and acuity**			
Female (%)	65.4 (10.9)	64.9 (12.0)	65.5 (9.1)
African American (%)	22.3 (25.7)	23.1 (26.2)	21.0 (26.3)
Moderate or severe cognitive impairment (%)	64.1 (11.8)	64.9 (9.1)	66.1 (11.8)
Potentially eligible residents (#)	44.8 (24.8)	44.7 (20.5)	45.3 (14.8)
Potentially eligible residents with agitated/aggressive behaviors (%)	20.1 (11.3)	20.5 (13.3)	20.5 (9.7)
Any antipsychotic use (%)	17.9 (8.6)	18.0 (8.3)	17.5 (12.0)
Total activities of daily living score^a^	16.7 (1.7)	16.5 (2.0)	16.9 (2.0)
**Nursing home quality, payment, and staffing**			
Total beds (#)	101.5 (42.3)	107.3 (40.0)	103.6 (33.0)
CMS^b^ 5-Star Quality Rating^b^	3.5 (1.4)	3.6 (1.2)	3.5 (1.2)
Medicaid as primary payer (%)	58.8 (25.6)	58.6 (27.6)	55.4 (26.1)
Medicare as primary payer (%)	11.2 (7.0)	11.5 (9.5)	11.1 (7.5)
Self-pay (%)	30.1 (26.4)	30.0 (24.7)	33.5 (28.5)
Registered nurse hours per resident day (#)	0.3 (0.2)	0.3 (0.2)	0.3 (0.2)
Licensed practical nurse hours per resident day (#)	0.9 (0.3)	0.9 (0.3)	0.8 (0.3)

*CMS* Centers for Medicare & Medicaid Services

^a^Describes the ability of the resident to perform activities of daily living. Higher scores indicate more dependence on staff

^b^Score ranges from 1 to 5 stars, with five stars indicating the highest quality nursing homes

### Blinding

{17a} Only aggregated post-random assignment comparisons of intervention and control NH’s baseline characteristics are viewed by the investigators. The study principal investigator is blinded to the identity of both the control and intervention NHs. {17b} Unblinding during a trial is not permissible.

### Sample size

{14} The required number of clusters to reach a pre-specified power was derived such that each of the two parallel trials is adequately powered to detect an effect size of *δ*. This may result in a conservative sample size estimation of the second parallel trial, because we do not consider the incorporation of the information from the first parallel trial in the sample size calculation. We collect information about resident’s CMAI score before and after the intervention was implemented for each of the two trials. Within each parallel trial, this design is referred to as a cluster-randomized trial with the pretest–posttest design [29]. It has been shown that by adjusting the posttest with the pretest score, the power of the study could be improved [29, 30]. To estimate the required sample size for different effect sizes, we used the formula proposed by Teerenstra et al. [30]. For significance level *α* and power 1-*β*, the formula for the required number of residents is:

$$ {n}_{res}=\frac{2{\left({Z}_{1-\frac{\alpha }{2}}+{Z}_{1-\beta}\right)}^2{\sigma}^2}{\delta^2}\left(1+\left(n-1\right)\rho \right)\left(1-{r}^2\right), $$

where *Z*_*x*_ is the critical value from a normal distribution at *x*, *σ*^2^ is the variance of the outcome CMAI, *δ* is the effect size, *ρ* is the intra-class correlation, *n* is the number of residents per cluster, and *r* is the correlation between a cluster means at baseline and at follow-up. To obtain the number of clusters required per arm, we would need *n*_*res*_/*n*. Assuming a nominal level of *α* = 0.05 and power of 80%, Table 3 describes the required number of clusters per arm for different effect sizes based on *n* = 15, *σ* = 20, *ρ* = 0.12, and *r* = 0.5. For a 6-point reduction in the total CMAI score, 24 NHs per study arm are required. To address possibly higher ICC values, non-participation, and lower correlation between the baseline and outcome scores, 27 NHs per study arm are recruited.

**Table 3:** Number of nursing homes needed for different effect sizes

Effect size^a^	4	6	8
Number of nursing homes needed per sequence^b^	53	24	13

^a^Difference in Cohen-Mansfield Agitation Inventory total score (primary study outcome)

^b^Assuming a nominal level of 0.05 and power of 80%

### Statistical methods

#### {20a, 20b, 20c} First parallel trial analysis

The analytic approach in the first parallel trial is based on the frequency of agitated and aggressive behaviors in a long-stay population with dementia after intended exposure to the intervention (treatment) or after 4 months (control), conditional upon survival to at least one post-intervention observation (up to 4 months after baseline measurement). {18b} Our primary analysis is based upon an intent-to-treat principle, and we estimate complier average causal effect as a secondary analysis. The complier analysis estimates the effects of the intervention for residents who received the music or would have received the music.

Our primary ITT analysis model is based on the model described by Murray and Blistein [29] and Teerenstra et al. [30]. Let *Y*_*ijk*_ be the staff interview for resident *i* ∈ {1, …, *n*} from NH *j* ∈ {1, …, *J*} at time *k* ∈ {*baseline*, *post* − *exposure*} and *X*_*ij*_ a set of baseline covariates for resident *i* from NH *j*. We assume that *Y*_*ijk*_ = *μ*_*ijk*_ + *ϵ*_*ijk*_, where ${\epsilon}_{ijk}\sim N\left(0,{\sigma}_{\epsilon}^2\right)$, and *μ*_*ijk*_ = *μ* + *α*_1_*I*_*ij*_ + *α*_2_*T*_*k* = 1_ + *θ*^*t*^*X*_*ij*_ + *δT*_*k* = 1_*I*_*ij*_ + *u*_*j*_ + (*uτ*)_*j*, *k*_ + *s*_*ij*_. We define *T*_*k* = *k*′_ to be an indicator function that is equal to 1 when *k* = *k*′ and 0 otherwise, ${u}_j\sim N\left(0,{\sigma}_u^2\right)$ is the deviation of cluster *j* from the overall mean, ${\left( u\tau \right)}_{j,k}\sim N\left(0,{\sigma}_{u\tau}^2\right)$ represent the variation of each cluster at different time points, ${s}_{ij}\sim N\left(0,{\sigma}_s^2\right)$is the variation of individuals, *I*_*ij*_ is an indicator for participating in the intervention group, *α*_1_ is the difference in baseline averages between control and treated units, *α*_2_ is the change from baseline to follow-up for the control cluster means, *θ*a vector of unknown coefficients, and *δ* is the conditional difference in change from baseline between intervention and control cluster means. The conditional treatment effect is then defined as *δ*. Individual-level covariates comprise baseline variables. The estimate of interest would be the difference in marginal means.

To estimate the effects among participants that would comply with the intervention, we used a technique described by Jo et al. [31]. Let *c*_*ij*_ be an indicator that is equal to 1 if resident *i* in NH *j* would use the music if provided. We assume that residents who would not be offered the music will not attempt to obtain it on their own. Eligible residents who do not receive the intervention and receive care in an intervention NH are referred to as “non-compliers.” The effects of the intervention would be estimated using, ${\mu}_{ij}={\beta}_0+{\beta}_c{c}_{ij}+{\alpha}_c{c}_{ij}{I}_{ij}+{\sum}_{l=1}^L{\gamma}_{ij l}{X}_{ij l}+{u}_{nbj}\left(1-{c}_{ij}\right)+{u}_{nwij}\left(1-{\mathrm{c}}_{\mathrm{ij}}\right)+{u}_{cwij}{c}_{ij}+{u}_{cbj}{c}_{ij}$, where the macro-unit residuals *u*_*nbj*_ (non-compliers) and *u*_*cbj*_ (compliers) represent cluster-specific effects given *I*_*ijk*_ and *X*_*ijl*_, which are assumed to be normally distributed with zero mean and the between-cluster variances *σ*^2^_*nb*_ (non-compliers) and *σ*^2^_*cb*_ (compliers), respectively. The micro-unit residuals *u*_*nwij*_ (non-compliers) and *u*_*cwij*_ (compliers) are assumed to be normally distributed with zero mean and the within-cluster variance *σ*^2^_*nw*_ (non-compliers) and *σ*^2^_*cw*_ (compliers) and are equal across clusters. The following model for compliance status was assumed:

$$ P\left({C}_{ij}=1\right)=\frac{\mathit{\exp}\left({\sum}_{l=1}^L{\pi}_{ij l}{X}_{ij l}+{\tau}_j\right)}{1+\mathit{\exp}\left({\sum}_{l=1}^L{\pi}_{ij l}{X}_{ij l}+{\tau}_j\right)} $$

where *π*_*ijl*_ are unknown parameters and ${\tau}_j\sim N\left(0,{\sigma}_{\tau}^2\right)$ so that the proportion of compliers may vary across clusters. Compliance status is only known in the intervention arm. Thus, a mixture model for compliance status in the control arm would be applied. Using the full likelihood, parameter estimates of the effect among compliers are estimated:

$$ \hat{\delta_c}=\left({\hat{\delta}}_{c1}-{\hat{\delta}}_{c0}\right)/{\rho}_c $$

where ${\hat{\delta}}_{ct}$ are the average CMAI among compliers in treatment group *t* and where *ρ*_*c*_ is the proportion of compliers. ${\hat{\delta}}_{ct}$ can be obtained from the above models across NHs. The variance of this estimate can be obtained via the delta method or using Markov chain Monte Carlo techniques.

#### Second parallel trial analysis

A similar model to the one described for the primary ITT analysis in the first parallel trial would be implemented in the second parallel trial. However, to gain efficiency among the control population, we would rely on the meta-analytic-predictive approach [32]. This approach assumes that model parameters for the control population of both trials are exchangeable and are drawn from the same distribution. In this trial, this assumption is appropriate, because all of the facilities were randomized at beginning of the trial and they are treating a similar population of patients. Using data on individuals that reside in control facilities in the first trial can be used to inform estimation of model parameters for individuals in control facilities in the second trial. This method was shown to achieve gain in precision while maintaining type I error [33].

### Data safety and monitoring

{21a} An independent data safety and monitoring board (DSMB) with no financial or other competing interests will act in an advisory capacity to the National Institute on Aging (NIA) Director to monitor participant safety, data quality, and progress of the study. {5d} The steering committee, consisting of the principal investigator (VM) and the project director (EM), will have ultimate responsibility for all aspects of the study, including ensuring timely submission of all requested project materials to the funder, serving as the primary liaison between the project and the NH corporations, coordinating tasks among individual working groups, ensuring project milestones are met, and reviewing and approving all publications. Members of the study team who will participate in the semi-annual sessions of the DSMB include the PI (VM), the lead biostatistician (RG), and the project director (EM). The NIA project officer will attend DSMB meetings and serve as the liaison between the DSMB and the funder.

### Timeline

{13} The study timeline is provided as Table 4.

**Table 4:** Timeline for the adaptive cluster-randomized trial

	Period 1 (June 2019–January 2020)	Period 2 (February 2020–April 2021)	Period 3 (June 2021–February 2022)
Sequence 1 (27 nursing homes)	Intervention*† (405 residents)	Use implementation data from period 1 to identify residents who are most likely to benefit from the intervention, to improve enrollment for period 3	
Sequence 2 (27 nursing homes)	Control*† (405 residents)		Intervention*† (405 residents)
Sequence 3 (27 nursing homes)			Control*† (405 residents

*Onsite primary data collection at baseline, 4 months, and 8 months, to interview staff about resident behaviors in the past week using the Cohen-Mansfield Agitated Inventory and to directly observed behaviors using Agitation Behavior Mapping Instrument

†Secondary data transferred monthly to capture agitated behaviors as reported in the Minimum Data Set and current medication orders as recorded in the electronic medical record

### Reporting harms

{22} The potential adverse events that could occur during this trial are distress or strong negative emotional reactions in response to the intervention or distress or strong negative emotional reactions in response to being observed. NH staff and data collectors are trained to report potential adverse events to the project director (EM). The project director will report potential adverse events to the PI (VM) via email or telephone immediately upon becoming aware of the event. All potential adverse events will be investigated and independently verified by the study geriatrician (JR). Verified adverse events will be reported quarterly to the Data Safety Monitoring Board (DSMB), the Program Officer, and the IRB. Unanticipated harms will be reported to the DSMB, the Program Officer, the Office for Human Research Protections, and the IRB within 24 h of the research team becoming aware of the event. {23} During verification, if it is determined that the event does not meet the criteria for an adverse event or unanticipated problem, the event reporting form and the event verification form will be retained for auditing purposes.

### Key design features of the adaptive trial

#### Test alternative implementation strategies

In a previous trial of M&M, a common implementation barrier cited was a lack of “buy-in” by nursing staff responsible for administering medications [34]. This lack of buy-in may, in part, result from a lack of nursing ownership early in the intervention. The training provided by M&M emphasizes the importance of identifying the songs the resident loved when s/he was a young adult [6]. To accomplish this, M&M recommends talking to family members and individually testing each song with the resident to look for a positive response. This time-consuming, trial and error process is typically completed by activity staff or volunteers [34]. Given activity staff work primarily during the day and do not administer medications, it is unlikely that they will be able to respond to behaviors in real time to reduce pro re nata (PRN) medication use.

However, personalization of the playlists is one of the core components [35] of the M&M intervention. In theory, the intervention works by eliciting memories triggered by music residents loved when they were young adults. There is some preliminary evidence to support that long-stored musical memories are retained into later dementia [7] and resident preferred music may provoke a more visceral reaction than calming music alone [36]. However, there is no evidence to suggest the degree of personalization that is necessary; does the music need to be the resident’s favorite songs or is familiarity sufficient? If familiarity is sufficient to calm behaviors, Spotify or similar streaming services would be a less time-consuming way to deliver the intervention.

To better understand the degree of personalization which is required to potentially affect behaviors in NH residents with dementia, we will test two implementation strategies separately in each of the two parallel trials. The first trial will use a full-personalized approach, in which activity staff test individual songs with residents to look for a positive reaction. Activity staff identify 25–50 songs that the resident appears to like, and the music player is then given to frontline nursing staff to use at early signs of agitation. The second trial will use a partially personalized strategy, in which nursing staff identify residents with behaviors who they think would benefit from the intervention. Then, research staff preload music players based on the demographics of the resident and his/her preferred genre (if known). Music players are sent directly to the nursing staff champion for use at early signs of agitation.

For each parallel trial, we will measure the degree of nursing engagement with the intervention by assessing the proportion of residents who are chosen for the intervention to address agitated behaviors and by asking nursing staff how often in the past week they have used the music with the resident. We will also measure the dose of music that is received under each approach. We will keep all the outcome measurements as close as possible in the two trials, while modifying the intervention delivery to better understand the importance of personalization on behavior and the effect of personalization on nursing use of the intervention.

#### Increasingly enroll residents who are likely to benefit from the intervention

Each participating NH is provided equipment for 15 residents to be exposed to the M&M program during the 8-month study period. Given that many sites have more than 15 potentially eligible residents, it is important to standardize the process for choosing residents. NHs in treatment and control arms of the parallel design are asked to select and rank order 15 residents to receive the intervention at baseline. Standardized guidance is provided to staff about how to choose and rank these residents. NHs are asked to start with residents who liked music, were visible to staff during the day, and had specific, non-severe behaviors. Early successes are key to moving forward with widespread intervention adoption.

At the intervention midpoint (4 months), NHs are allowed to replace residents from their original lists who had died or been discharged from the NH. At this point, there is a potential for differential selection of replacements between treatment and control NHs because treatment NHs have been using the intervention and learning what type of residents seem to most benefit from the intervention. During the year between parallel trials, we will examine this selection process as well as play data from the music devices to identify resident demographic and clinical characteristics associated with greater use of the music devices and greater likelihood of being chosen by NH staff at the intervention midpoint. At the beginning of the second trial, we will use this information to help NH staff better choose residents who are likely to benefit from the program, a hallmark of adaptive trials [37].

#### Seamlessly conduct a combination stage III/IV trial

This study was originally designed as a stage IV embedded pragmatic trial (ePCT) [16], a hallmark of which are case and outcome ascertainment using available data sources (MDS and EHR) [38, 39]. However, during the pilot phase of this research, we found considerable under-detection of behaviors in the MDS data [23], raising questions about the sensitivity of MDS data to detect changes in behaviors resulting from the music intervention [26]. The protocol was altered to have researchers visit NHs and collect “gold standard” CMAI measures in addition to the NH-collected measure.

The CMAI and ABMI require researchers to visit NHs, observe residents, and interview staff, an expensive proposition for researchers and a less pragmatic approach than using existing data. To compensate for this under ascertainment, we will develop a statistical measurement model to equilibrate the MDS-ARBS to the CMAI and ABMI resident behavioral data among the treated and control NHs using the complete data set during the first trial. This model will be validated using data from the second trial.

This statistical imputation model will be used two ways. First, we will use the imputation model to address missingness of baseline CMAI in the current trials. Using the estimated relationship between instruments, CMAI and ABMI scores will be multiply imputed for residents for whom only the MDS-ARBS is available [40, 41]. We will rely upon a two-stage imputation procedure allowing us to compare all residents using common instruments, increasing the efficiency of the study design because these two measures are known to be reasonably correlated [42]. Formally, the multivariate ordinal probit model will be used to estimate the relationship between the three different scales (CMAI, ABMI, MDS-ARBS) while adjusting for demographics and other characteristics (e.g., gender, race, physical function, and comorbidities) [41]. Using estimates from these models, CMAI and ABMI will be multiply imputed for residents who are missing a baseline or outcome measures. This will result in *K* multiple datasets for which CMAI and the results would be combined using common combination rules [43].

Second, we will consider the generalizability to future pragmatic trials of non-pharmaceutical interventions for NH residents with dementia. If we demonstrate that our imputation model is relatively accurate, other researchers could use this model to generate a more sensitive score that can be used in large-scale pragmatic trials of non-drug interventions in this population. This would allow for cost-effective, large-scale evaluation when an intervention lacks effectiveness evidence and simple application of available administrative measures may not be appropriately sensitive.

## Discussion

Using an adaptive study design, we are conducting two parallel, cluster-randomized controlled trials. The adaptive design has three key features: test alternative implementation strategies, increasingly enroll residents who are likely to benefit from the intervention, and seamlessly conduct a stage III/IV trial. To our knowledge, this is the first cluster-randomized trial to utilize an adaptive study design.

The proposed adaptive design has the potential to reduce the research timeline by leveraging enrollment and recruitment for one large study to test two implementation strategies. The current best-practice M&M protocol involves full personalization of the music playlists through individual testing of the songs with the residents with dementia to look for a positive response [6]. While there is some evidence to support that early learned music is better for recall than late learned music [7], and preferred music is better than “calming” music [36], there is no evidence on how personalized the music playlists need to be. The only existing trial of the existing best-practice protocol is small (59 residents) with low adherence (music was used an average of 9 days a month) [14]. The next step of this research is test the same protocol with an adequate sample and increased adherence monitoring. However, qualitative work from the same study suggested that the process for identifying resident preferred music was time-consuming and potentially a barrier to use [34]. The adaptive trial design allows us to test the existing protocol in a larger trial with increased adherence monitoring and to conduct a subsequent trial with a partially personalized music playlist strategy. If partial personalization is sufficient, the intervention could be more readily implemented by nursing staff, which is likely to result in more substitution of the intervention for PRN medications.

Another benefit of this design is that it allows us to better identify who is likely to benefit from the intervention and test that hypothesis within the same trial. Often, we are forced to rely on post hoc subgroup analyses to describe populations who are most likely to be affected by the intervention. These types of analyses are hypothesis generating at best and can lead to spurious results which are often underpowered [44–46]. In this adaptive trial, we will use an observed selection from the first trial as well as play data from the music devices to identify resident demographic and clinical characteristics associated with greater use of the music devices and greater likelihood of being chosen by NH staff. We will use this information to guide NH staff on the choice of residents who are likely to benefit from the program for the second trial. As the number and type of sensory and reminiscence therapies for people with dementia grow [47], it is important to be able to identify which non-pharmaceutical alternatives are likely to work for specific individuals [48]. This adaptive feature has the potential to help us better match available interventions to residents.

The combined stage III/IV feature of the adaptive trial design has the potential to produce a scalable, cost-effective solution for dealing with under-detection of outcomes in administrative data. Using routinely collected administrative data to assess outcomes for participants is one way to increase pragmatism in study eligibility and contain study costs [49, 50]. However, administrative data have known biases. In the case of behavioral data, our primary outcome, NH staff normalize the behaviors of residents that they interact with every day and only document the most severe behaviors leading to under-detection in the associated measures [51]. By equating on-site researcher-collected data to available NH-collected administrative data at the resident level, we can derive a more sensitive behavioral score using available administrative data without on-site data collection.

For this trial, we originally proposed a stepped wedge design in which 81 NHs received the intervention over the course of 3 study years (27 NHs per year). Enrollment of residents for the first study year began in June 2019 and ended in January 2020. We were forced to pause the training and roll-out of the intervention in NHs randomized to receive the intervention in the second study year because of the emergency response to the coronavirus pandemic in nursing homes. The stepped wedge trial design is sensitive to confounding by time, particularly when time is correlated with the study outcome due to a secular trend (like the increased agitation which may well have occurred during a national pandemic) [52]. Thus, we believed that the use of a stepped wedge design to complete the remainder of the study was irreparably damaged by this exogenous shock. We revised our study protocol to include the use of an adaptive trial design to conduct two parallel trials. This modified trial protocol was approved by the National Institute on Aging and an independent data safety and monitoring board in December 2020.

This trial has limitations. Interventionists traditionally establish efficacy before testing effectiveness using pragmatic methods [16, 39]. Yet there may be valid reasons to test interventions with limited efficacy under real-world conditions—for example, when there are populations or settings in which it is not possible to obtain traditional efficacy data [53]. We decided to proceed with this trial, in part because there is a pressing need for effective non-pharmaceutical interventions to address dementia-related behaviors in NHs and because traditional efficacy studies systematically fail to enroll complex populations and typically require proxy for consent [54, 55]. Residents with involved proxies differ from typical residents with dementia in important ways, including race [56], that may affect consent and the generalizability of efficacy studies. In such instances, it may be important to accelerate the testing of promising interventions. There are also several characteristics of this trial design which are not fully pragmatic. The PRECIS-2 tool assists researchers to identify and justify the level of pragmatism of their study along nine relevant domains [38]. Our trial is highly pragmatic in six of the nine trial domains (recruitment, setting, delivery, adherence, outcome, and analysis), reflecting the flexibility of real-world implementation and the primary intent-to-treat analysis. The trial is less pragmatic in three PRECIS-2 domains—follow-up, organization, and eligibility. Our deviations from full pragmatism are direct results of piloting our implementation, measurement, and recruitment strategies. We argue that fully pragmatic trials are rare [57, 58], and piloting helps researchers understand where compromises must be made along the explanatory–pragmatic continuum to maintain the integrity of the research [59].

This design has the potential to reduce the research timeline by leveraging enrollment and recruitment for one large study to test two implementation strategies, increasingly enroll residents who are likely to benefit from the intervention, and addressing known limitations associated with using administrative data to evaluate behavioral outcomes. Similar approaches may be of interest to funders, researchers, and clinicians serving populations in need of timely solutions to real-world problems.

## Supplementary Information

**Additional file 1 **SPIRIT Checklist for *Trials*.

## Data Availability

The datasets generated from the proposed study will be made available in the Brown University data repository: https://repository.library.brown.edu.
